# Multimodal Therapy of Locally-Advanced Penile Cancer: A Case Report With Literature Review

**DOI:** 10.7759/cureus.57163

**Published:** 2024-03-29

**Authors:** Meriem Bouabid, Souad Margoum, Ahmed BenSghier, Nadir Miry, Amal Bennani, Mohamed Moukhlissi, Soufiane Berhili, Loubna Mezouar

**Affiliations:** 1 Department of Radiation Oncology, Centre Hospitalier Universitaire Mohammed VI, Oujda, MAR; 2 Department of Pathology, Centre Hospitalier Universitaire Mohammed VI, Oujda, MAR

**Keywords:** prognosis, radiotherapy, chemotherapy, surgery, penile cancer

## Abstract

Cancer of the penis is a rare tumor that occurs in the elderly. Because of its rarity, it is often not diagnosed early, and its treatment poses difficulties for practicing oncologists. We report the case of an elderly patient treated for locally advanced squamous cell carcinoma (SCC) of the penis, with a review of the literature. A 71-year-old man, who had been complaining of pruritus on the penis two years ago, presented with an ulcerated lesion on the prepuce and the glans. A biopsy of the lesion with pathological study showed a SCC of the penis. Pelvic MRI showed tumor thickening centered on the glans of the penis, infiltrating the fascia and the spongy urethra with discrete upstream dilatation and bilateral inguinal adenomegaly. CT scan of the neck, chest, abdomen, and pelvis showed no secondary localizations. Treatment initially consisted of carcinological surgery by a partial penectomy with bilateral inguinal lymph node dissection. The tumor was therefore classified as pT3N3M0. A PET CT scan performed later was in favor of local and regional recurrence. Surgery was not feasible, so concomitant chemo-radiotherapy was indicated at a total dose of 70 Gy in 35 fractions of 2 Gy concomitantly with platinum-based chemotherapy, withgood evolution.

## Introduction

Penile cancer is a rare disease. Its incidence varies according to the geographical localization. It's much frequent in South America, Southeast Asia, and parts of Africa accounting for 1-2% of malignant tumors. The incidence increases with age with a peak at 60 years. Because of its rarity, on the one hand, it is often not diagnosed early, and on the other, its treatment poses difficulties for practicing oncologists [1,2].

The main risk factor is the human papillomavirus (HPV) infection. There are other factors including phimosis, chronic penile inflammation, ultraviolet radiation, smoking, and low socioeconomic status [3].

 We report the case of a patient treated at the Hassan II Oncology Center of Oujda for cancer of the penis, with a review of the literature to assess the epidemiological, clinical, therapeutic, and prognostic features of this pathology.

## Case presentation

**Figure 1:**
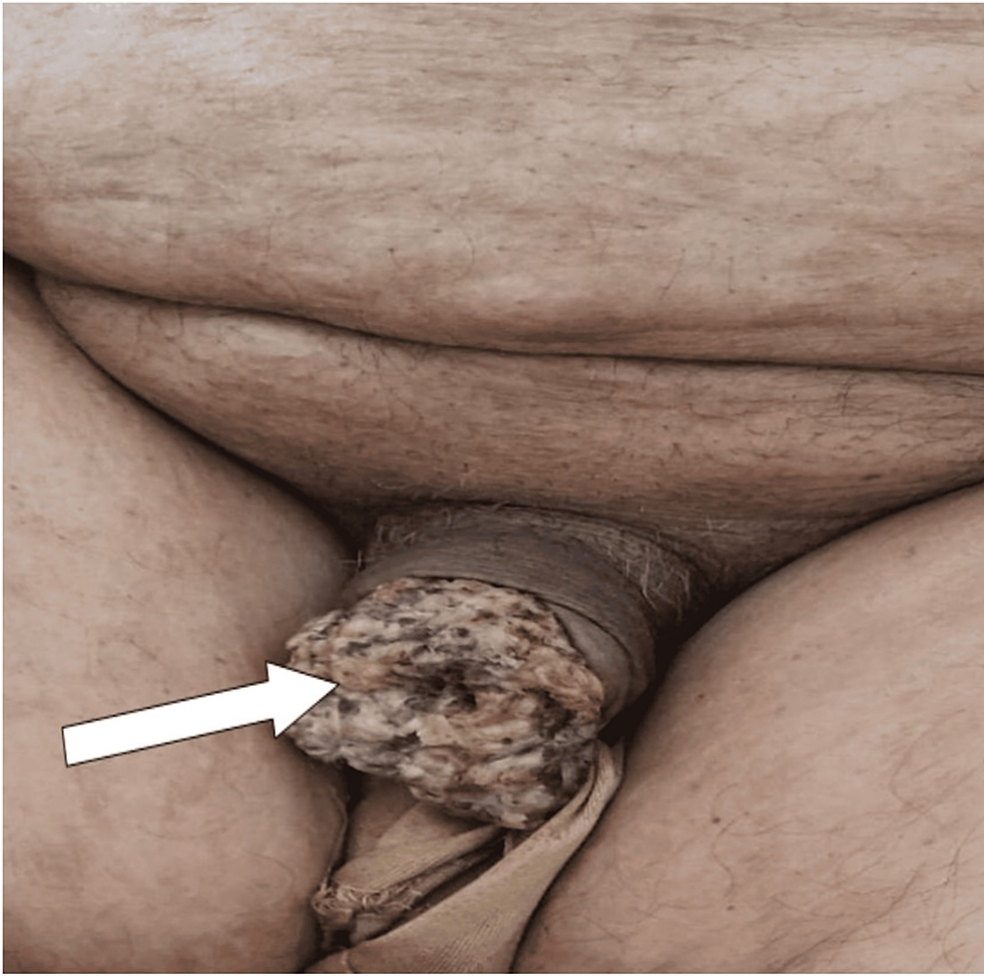
Ulcerated lesion of the penis, localized on the prepuce and the glans (white arrow)

The patient was a 71-year-old diabetic who had been complaining of pruritus on the penis two years ago, with the appearance of an ulcerated lesion on the prepuce and the glans, progressively increasing in size, with no other associated signs (Figure 1).

**Figure 2:**
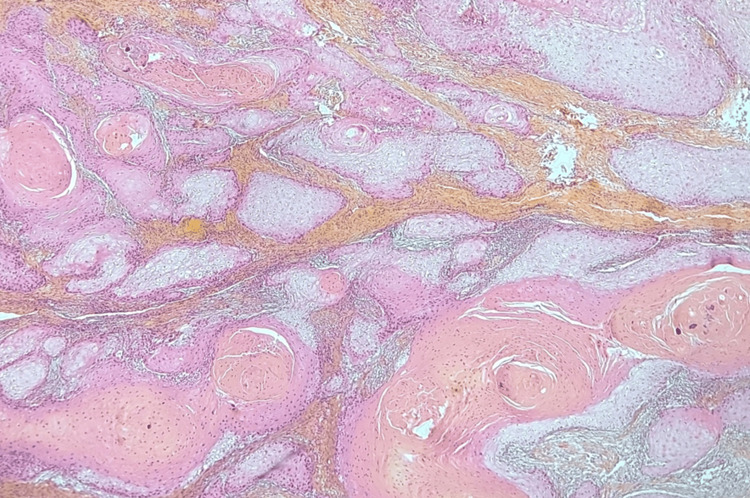
Photomicrograph of the lesion showing multiple nests of well-differentiated squamous cell carcinoma displaying abundant keratinization (HES, x40) HES: hematoxylin-eosin saffron stain

**Figure 3:**
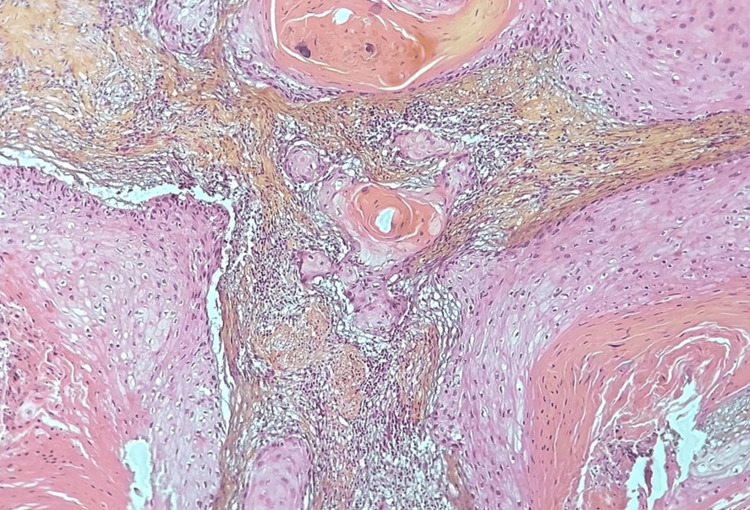
At higher magnification, tumor cells are polygonal, showing mild atypia with round nuclei and abundant eosinophilic cytoplasm (HES, x200) HES: hematoxylin-eosin saffron stain

A biopsy of the lesion with pathological study was done which showed a squamous cell carcinoma (SCC) of the penis (Figures 2, 3).

**Figure 4:**
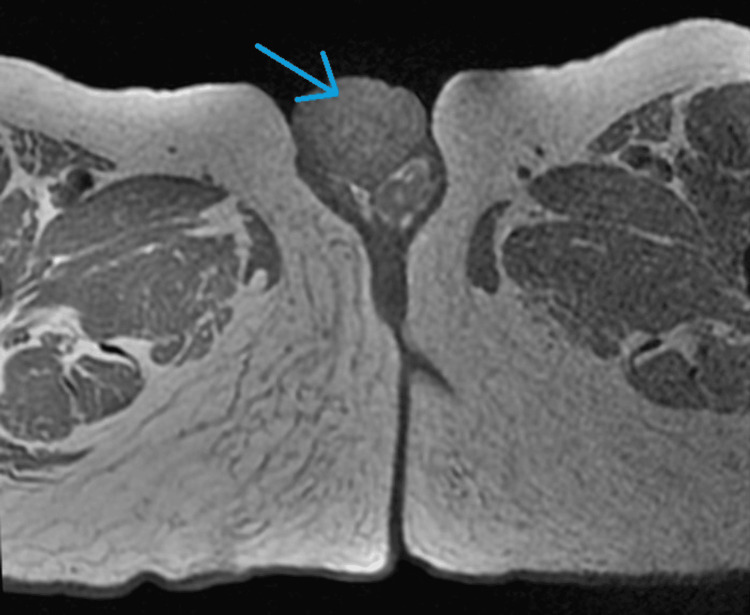
Axial pelvic MRI T1 showing tumor thickening centered on the glans of the penis (blue arrow)

**Figure 5:**
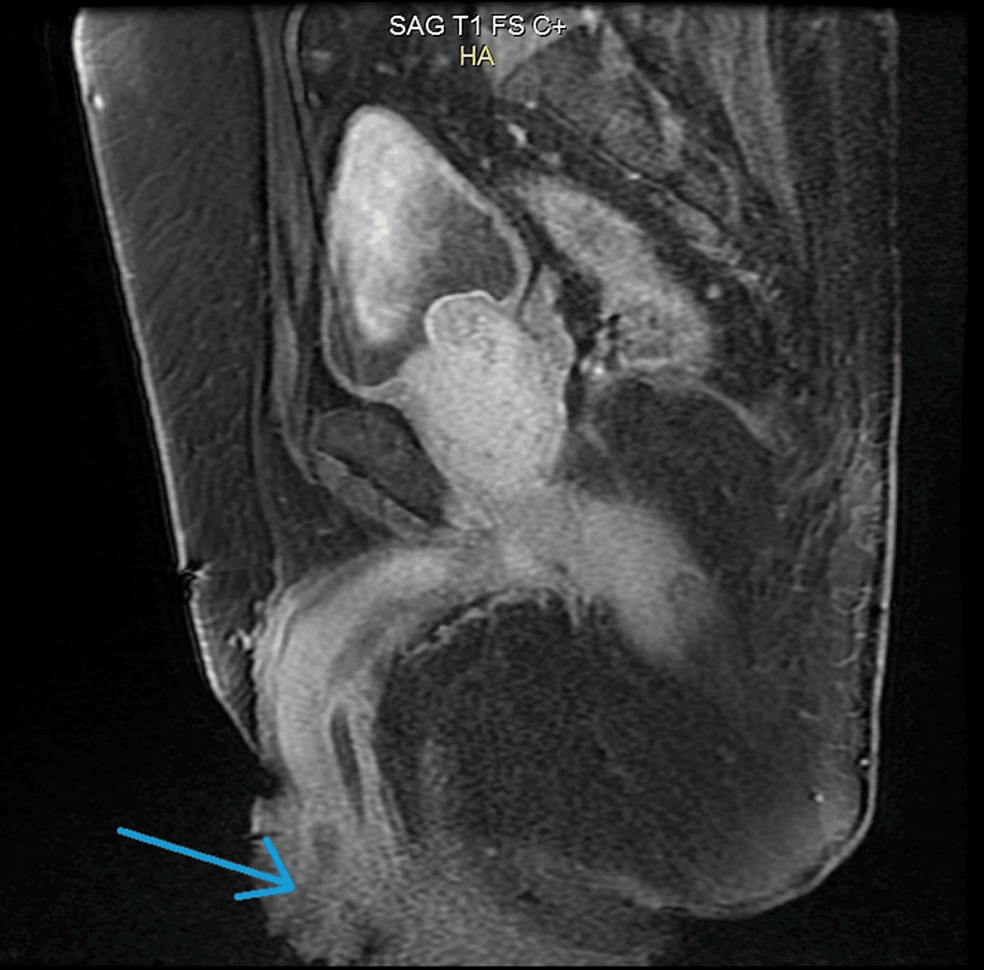
Sagital pelvic MRI T1 showing tumor thickening centered on the glans of the penis (blue arrow), infiltrating the fascia and the spongy urethra

**Figure 6:**
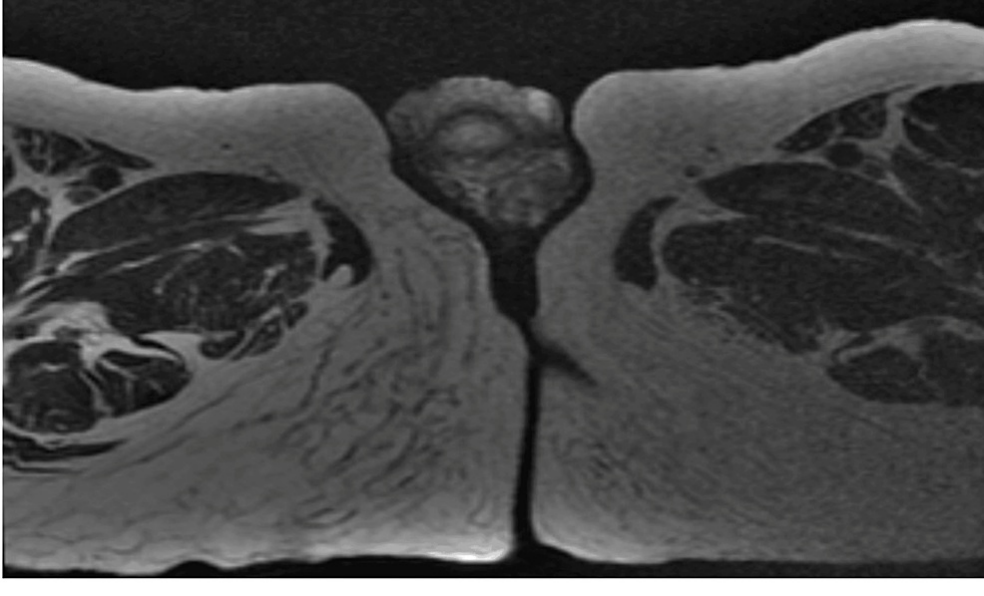
Axial pelvic MRI T2 showing tumor thickening centered on the glans of the penis, infiltrating the fascia and the spongy urethra

**Figure 7:**
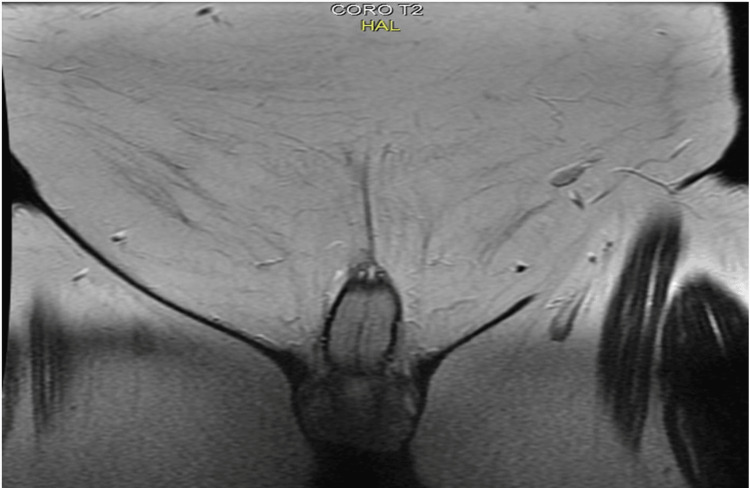
Coronal pelvic MRI T2 showing tumor thickening centered on the glans of the penis, infiltrating the fascia and the spongy urethra with discrete upstream dilatation and bilateral inguinal adenomegaly

Pelvic MRI (Figure 4-7) showed tumor thickening centered on the glans of the penis, infiltrating the fascia and the spongy urethra with discrete upstream dilatation and bilateral inguinal adenomegaly. CT scan of the neck, chest, abdomen, and pelvis showed no secondary localizations.

Treatment initially consisted of carcinological surgery by a partial penectomy with bilateral inguinal lymph node dissection. Histopathology revealed a well-differentiated keratinizing and infiltrating SCC. The cavernous and the spongy body of the penis, as well as the urethral duct, were infiltrated by the tumor. The limits of resection were negative, passing 9 mm from the cutaneous border, 16 mm from the urethral border, and over 20 mm from the cavernous and spongy borders. Inguinal lymph node dissection found 5 N+/13 N (1 capsular effraction) on the left, whereas no lymph node parenchyma was found on the right. The tumor was therefore classified as pT3N3M0.

**Figure 8:**
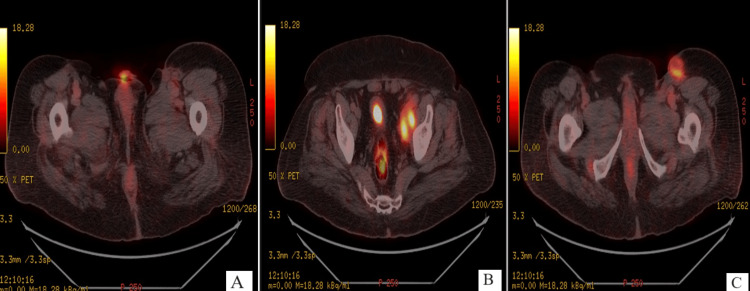
PET CT scan with a hypermetabolic focus in the stump of the penis (A, SUVmax of 7.5) in favor of local recurrence, with extensive pathological active lymph node involvement in left pelvic lymph node (B, SUVmax of 7.9) and in the left inguinal lymph nodes (C, SUVmax of 6.4) SUVmax: standardized uptake value

When the patient was admitted to the radiotherapy department, clinical examination noted the appearance of a suspicious nodule in the left inguinal lymph node area, suggestive of tumor recurrence. A PET CT scan (Figure 8) was then performed, showing extensive pathological active lymph node involvement in the left inguinal (standardized uptake value (SUVmax) of 6.4) and left pelvic (SUVmax of 7.9) lymph nodes, with a hypermetabolic focus in the stump of the penis (SUVmax of 7.5), in favor of local and regional recurrence.

Given the significant locoregional extent of the recurrence, surgery was not an option, so concomitant chemo-radiotherapy was indicated at a total dose of 70 Gy in 35 fractions of 2 Gy concomitantly with platinum-based chemotherapy (cisplatin) at a dose of 40 mg/m^2^/week.

Radiotherapy went well, with some mild side effects (grade II radiodermatitis), which improved with medical treatment. The patient had a single episode of acute urinary retention, for which a urinary catheter was done. Every 15 days, the patient underwent urethral dilatation in the urology department to prevent urethral obstruction. The post-irradiation check-up showed a good clinical and radiological evolution. The patient was free of recurrence at the six-month follow-up.

## Discussion

Cancer of the penis often manifests itself as a papillary lesion or as an ulcero-infiltrative lesion that is rapidly associated with lymph node invasion. It often arises from the glans or prepuce [3,4]. Penile MRI is the most sensitive examination, providing a better assessment of any infiltration of the corpus cavernosum penis, and can help to determine whether conservative surgery is possible [5]. Biopsy with pathological study is done to confirm the diagnosis of SCC of the penis, which is the most frequent type. There are other rarer forms such as melanoma, basal cell carcinoma, and sarcoma [6].

The therapeutic approach to penile cancer has two fundamental aspects: oncological and functional, the latter being fundamental to micturition and sexual function. Additionally, there is the psychological aspect, which affects body image and self-esteem [7]. In general, local conservative non-invasive treatment is preferred for localized tumors. However, in the case of locally advanced tumors, as in the current case, partial or total penectomy with bilateral pelvic lymph node dissection is indicated [5,8,9].

Adjuvant treatment with concomitant radiochemotherapy is realized in the case of positive surgical margins and pelvic or inguinal lymph node invasion and also tumor recurrence at a total dose of 66-70 Gy, 2 Gy by fraction concomitantly with chemotherapy by cisplatin 40 mg/m^2^/week [10]. Therapeutic strategies for cancer of the penis can be extrapolated from studies on cancer of the vulva. Trials of chemoradiotherapy have good response rates and improve overall survival [11].

The International Penile Advanced Cancer Trial (InPACT) is the first phase III trial of advanced penile cancer. It is studying the benefits of surgery, chemotherapy, and chemoradiotherapy, and will provide important guidelines on treatment for penile cancer [12].

According to White et al., adjuvant multimodal therapy should be done for patients with high-risk features, principally pelvic lymph node metastases, extranodal extension, bilateral inguinal lymph node involvement, and tumor size >4 cm [13]. According to recent recommendations of the European Association of Urology (EAU), the optimal indication and order for multimodal treatment strategies for penile cancer with loco-regional lymph node involvement is difficult to discern. Therefore, adjuvant therapy with radiotherapy, chemotherapy, or chemo-radiotherapy provides great benefits in pN2-3 disease [14].

Survival in penile cancer depends on the disease stage and principally on lymph node invasion, which is the main prognostic factor. The five-year overall survival is approximately 85% but is only around 40% if lymph node invasion occurs [15,16].

## Conclusions

Penile cancer is a rare malignant tumor; its prognosis depends mainly on lymph node involvement. In patients with advanced penile cancer, there must be a multidisciplinary approach to treatment, including experts in urological surgery, radiation oncology, and medical oncology, in order to improve local disease control and long-term survival.
